# Twin-compartment solid–liquid cells for neutron reflectometry

**DOI:** 10.1107/S1600576726000919

**Published:** 2026-03-08

**Authors:** Nicoló Paracini, Hannah Burrall, Thomas Saerbeck, Philipp Gutfreund, Giovanna Fragneto, Luke A. Clifton, Thomas Arnold, Marité Cárdenas

**Affiliations:** a Department of Pharmacy, Faculty of Health and Medical Sciences, University of Copenhagen, 2100Copenhagen, Denmark; b European Spallation Source ERIC, Data Management and Software Centre, Asmussens Allé 305, 2800Lyngby, Denmark; c European Spallation Source ERIC PO Box 176 SE-221 00 Lund Sweden; d Institut Laue–Langevin ILL DS/LSS 71 Avenue des Martyrs F-38000 Grenoble France; e ISIS Pulsed Neutron and Muon Source, Science and Technology Facilities Council, Rutherford Appleton Laboratory, Harwell Science and Innovation Campus, Didcot, Oxfordshire OX11 OQX, United Kingdom; f Biofisika Institute, Science Park of the UPV/EHU, Barrio Sarriena s/n, 48940 Leioa, Spain; g Ikerbasque Research Foundation, Bilbao, Spain; h Department of Biomedical Sciences, Malmö University, Malmö 20506, Sweden; Lund University, Sweden

**Keywords:** solid–liquid cells, sample environments, neutron reflectometry, solid–liquid interfaces, thin films

## Abstract

We propose a new type of sample environment for neutron reflectometry at solid–liquid interfaces. The twin-compartment solid–liquid cell optimizes several aspects of neutron reflectometry measurements by splitting the surface of substrates currently in use at facilities into two independent samples. This approach increases the capacity of mounted samples on the stage of current neutron reflectometers by a factor of two, shortens alignment times, and enables side-by-side comparison and control experiments on identical substrates. Furthermore, it reduces sample preparation time for a variety of samples prepared by Langmuir monolayer transfer techniques, spin coating, sputter coating, *ex situ* self-assembly and more. We present results from three cell prototypes tested on vertical and horizontal reflectometers at ISIS and Institut Laue–Langevin.

## Abstract 2

We introduce a twin-compartment solid–liquid cell for neutron reflectometry that divides a single substrate into two independent measurement areas, doubling sample capacity and enabling direct side-by-side comparisons. This sample environment also reduces alignment and sample preparation time and has been successfully tested on both vertical and horizontal reflectometers at ISIS and Institut Laue–Langevin.

## 1. Introduction

Neutron reflectometry (NR) is a versatile technique for the structural characterization of a wide range of interfaces of scientific and technological interest (Skoda, 2019 ▸). The sub-nanometre spatial resolution along the direction perpendicular to the interface plane grants access to structural information that remains challenging to obtain with other methods. The two reflectometers under construction at the European Spallation Source (ESS) – Freia (horizontal sample plane geometry optimized for free liquids) and Estia (vertical sample plane geometry optimized for small and magnetic samples) – are designed to deliver high-flux neutron beams set to decrease measurement times and accessible sample sizes (Andersen *et al.*, 2020 ▸). In order to take advantage of the capabilities of these next-generation instruments, a concomitant development is required in the available sample environment typically used in reflectometry experiments.

In the scattering community, the term ‘sample environment’ refers to a wide range of auxiliary equipment used in experiments to house the sample in the beam and control conditions such as temperature, humidity, magnetic and electric fields, and so on (Le Brun & Gilbert, 2024 ▸). One of the most popular types of sample environment used in NR experiments today are solid–liquid cells. These are used to probe the interface between a solid, typically a large silicon crystal, and a liquid phase. The silicon substrates employed at European facilities such as ISIS and Institut Laue–Langevin (ILL) are primarily rectangular blocks of typical dimensions 50 × 80 × 15 mm^3^, whereas cylindrical sections with a diameter between 50 and 100 mm and a 5–10 mm thickness are used in the USA, at SNS (https://neutrons.ornl.gov/lr) and NCNR (Dura *et al.*, 2017 ▸), and in Australia at ANSTO (Le Brun *et al.*, 2023 ▸). Round wafers are better suited for spin coating because their circular symmetry tends to produce more uniform films, and they are often more readily available because they align with standard silicon manufacturing in which single-crystal ingots are grown as cylinders and then sliced into wafers. By contrast rectangular substrates can better match the typically rectangular neutron-beam footprint. Silicon crystals are the most popular substrates for solid–liquid NR because of their high neutron transmission and low incoherent scattering, which enables reflectivity measurements to be performed with the impinging beam approaching the interface through the silicon side. Crystalline silicon has the advantage of being a widely available substrate that can be reliably polished to a root mean square roughness of <4 Å, a key requirement for high-quality reflectivity data. Furthermore, surface modification via silane chemistry provides a versatile approach to obtain functionalized interfaces for a broad range of science cases (Wang *et al.*, 2021 ▸; Choi *et al.*, 2022 ▸). Silicon can also be coated with a range of thin metal layers, including gold, which enables electrochemistry experiments and a wide range of functionalizations through thiol–gold chemistry (Clifton *et al.*, 2019*b* ▸). Coating silicon with magnetic alloys enables application of magnetic contrast NR (Zubayer *et al.*, 2025 ▸); these alloy layers can be combined with additional surface coatings to form a magnetic buried layer (Holt *et al.*, 2009 ▸; Hughes *et al.*, 2014 ▸).

A solid–liquid cell for NR experiments with rectangular substrates is typically composed of two parts that clamp the substrate and create a water-tight reservoir between the polished solid face and one of the two halves of the cell, which is sealed with the substrate by an o-ring and connected to inlet and outlet valves by Teflon tubing (Welbourn & Clarke, 2019 ▸; Clifton *et al.*, 2019*a* ▸). The liquid injected through the inlet tubing flows in the plumbing channels of the solid–liquid cell and delivers the solution to one or more apertures distributed along the sides of the reservoir, filling the cell and flowing through the outlet on the opposite side. The approach that uses cylindrical wafers is based instead on clamping together two silicon wafers separated by a thin gasket which defines the volume of the reservoir, in the range of 50–200 µm in thickness. The silicon wafer acting as the backplate presents a roughened surface facing the liquid that avoids reflections from the additional silicon/water interface created (Hoogerheide *et al.*, 2022 ▸; Hoogerheide *et al.*, 2020 ▸). Alternatively, in electrochemical cells that employ a lithium counter-electrode on the backplate, a sufficiently thick lithium layer acts as an absorber which prevents additional reflections (Dura *et al.*, 2017 ▸; Rus & Dura, 2019 ▸).

**Figure 1:**
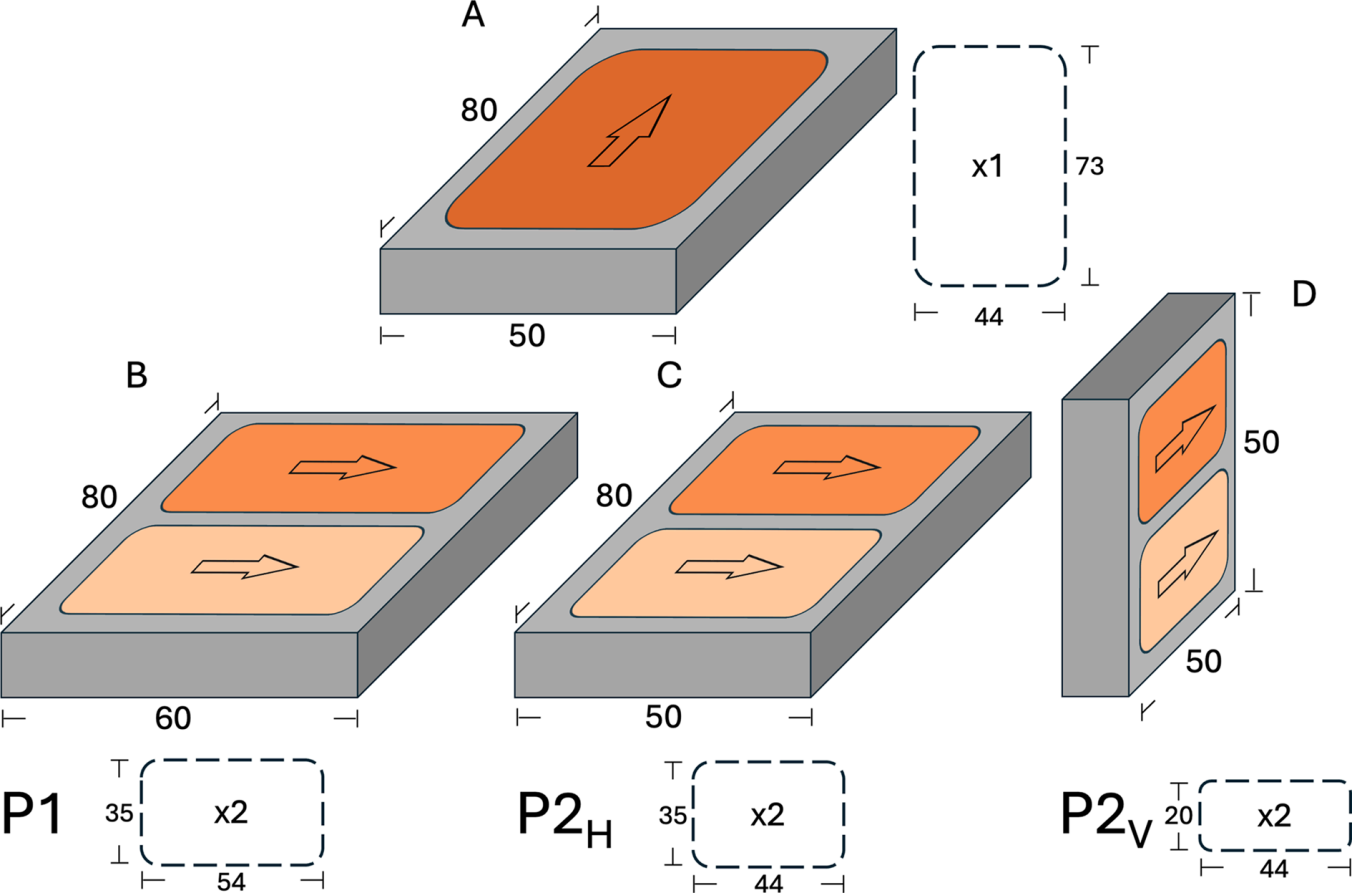
Split substrate approach of the twin-compartment cell. Schematics of footprints of solid–liquid compartments (orange) on silicon blocks (grey). Single-compartment cell (A) and twin-compartment cells tested here (B, C and D). The length and width of the silicon crystals (in mm) correspond to the size of the substrates used in the respective prototypes tested in this work, and arrows indicate the direction of the neutron beam. The dashed rectangles illustrate the compartment sizes measured from the centre of the O-ring grooves. The thickness of the reservoir in P2_H_ and P2_V_ is 0.35 mm, which yields a volume of ∼500 and ∼300 µl in the two models, respectively. In these models, the injection channels and distribution grooves add ∼250 µl to the volume of each reservoir.

Historically, NR has been a flux-limited technique that relied on large illuminated sample areas of several tens of cm^2^ in order to maximize the available beam footprint on the sample. However, since the first measurement of a quartz/water interface on D17 at ILL by Lee *et al.* (1989 ▸), beamline upgrades and brighter sources at facilities around the world have increased the performance of neutron reflectometers dramatically. This has enabled single-shot time-of-flight reflectivity measurements as fast as 120 ms (Saerbeck *et al.*, 2018 ▸) as well as low-noise measurements extending the useful *Q* range to ∼0.5 Å^−1^ (Hoogerheide *et al.*, 2022 ▸). Some of the approaches that have been proposed and implemented to increase the performance of reflectometers include the widespread installation of improved neutron transport guides at many facilities; new detector technologies, such as ^10^B-based Multi-Blade detectors (Piscitelli *et al.*, 2024 ▸), ^6^Li-based pixelated detectors (Chong *et al.*, 2022 ▸), HOPG-based energy dispersive detectors (Maliszewskyj *et al.*, 2018 ▸) and wavelength-shifting fibre-based detectors (Mauri *et al.*, 2021 ▸); and novel approaches to reflectometry exploiting divergent-beam geometries (Cubitt *et al.*, 2015 ▸; Stahn & Glavic, 2016 ▸; Stahn *et al.*, 2012 ▸) and polychromatic (Hoogerheide *et al.*, 2022 ▸; Maliszewskyj *et al.*, 2018 ▸) and white beams (Cubitt *et al.*, 2018 ▸). These enhanced capabilities not only enable faster measurements but also relax requirements for the large sample sizes typically required for NR samples. This reduction in sample surface area has so far primarily benefited the hard-matter community, as it enables the measurement of small surfaces down to a size of <1 cm^2^ (Callori *et al.*, 2020 ▸; Jochum *et al.*, 2019 ▸; Zhang *et al.*, 2021 ▸; Stahn & Glavic, 2016 ▸). In this context this capability is highly desirable given that lateral homogeneity is challenging to achieve over large areas for epitaxial films. In the new landscape of high-performance neutron reflectometers there are several advantages that can be harvested by the soft-matter community by rethinking the design of solid–liquid cells to optimize measurements of smaller surface areas. In this sense, there are two approaches that can be taken. The first is to simply shrink the size of all the components of the current design of a solid–liquid cell, which has, in its concept, remained the same since the first experiments in the late 1980s. This would mean a smaller cell, housing a smaller substrate to study a smaller sample. In this paper, we describe a second, different way to look at the problem by running NR experiments on a twin-compartment solid–liquid cell. This enables the formation of two individual samples on a single substrate of the dimensions currently in use at facilities like ISIS and ILL (Fig. 1 ▸).

The primary advantage of dealing with a reduced sample surface in a solid–liquid experiment is the smaller amount of solution required to fill the chamber of the cell. Smaller reservoirs consume less D_2_O but most importantly require smaller volumes of scarce and precious deuterated and biological materials, often available only in very limited quantities. This is a common problem with proteins, lipids and other biomacromolecules, which can be difficult to produce in sufficiently large amounts to fill current solid–liquid cells yet are often studied by NR (Haertlein *et al.*, 2016 ▸; Duff *et al.*, 2022 ▸; Paracini *et al.*, 2020 ▸; Lakey *et al.*, 2022 ▸). The same applies to other deuterated compounds often studied by NR, including polymers (Torikai *et al.*, 2007 ▸; Li *et al.*, 2021 ▸; Hafner *et al.*, 2021*a* ▸; Hafner *et al.*, 2021*b* ▸).

In this context, the twin-compartment approach offers several additional advantages over a more conventional miniaturization strategy: (i) An increase in the number of samples mounted on the sample stage by a factor of 2. A neutron reflectometer can usually accommodate up to six solid–liquid cells on the sample stage, which are positioned in the beam by lateral translation. Using two-compartment cells maintains the same number and arrangement of magnetic kinematic mounts in use today to anchor the current design of solid–liquid cells while effectively doubling the number of experiments that can be set up on the beamline at one time. (ii) An efficient use of substrates currently available at existing facilities. The two designs of solid–liquid cells described here house the two current most popular substrate sizes in use at ISIS and ILL: 80 × 50 mm^2^ and 50 × 50 mm^2^. (iii) A gain in alignment time. Aligning one compartment automatically aligns the second compartment, saving time required for setting up measurements. (iv) A reduction in the time spent characterizing substrate. Measuring one side of the cell already provides information on the other compartment, assuming the substrate’s structure is equivalent on both sides. (v) Side-by-side comparison experiments on the same substrate, including pre-functionalized surfaces. (vi) Reduced sample preparation time for a variety of sample types prepared *ex situ* (*e.g.* samples prepared by spin coating, Langmuir monolayer transfer techniques, nanolithography, sputter coating, *ex situ* self-assembly and so on). (vii) Reduced cleaning times of the cells and substrates. (viii) Increased accuracy of data analysis. Parameters related to the substrate structure can be shared between the samples prepared in the two compartments assuming the substrate’s structure is equivalent on both sides.

Here, we manufactured and tested the performance of three prototypes, named P1, P2_H_ and P2_V_, of the novel twin-compartment cell design. We used the cells in NR experiments performed at ISIS and at the ILL. The results show that this cell design optimizes several aspects of the sample preparation, measurement and data analysis whilst drastically reducing the solution volumes required to exchange the solvent inside the cells, which show complete exchange with volumes as low as 1.7 ml.

## 2. Materials and methods

### 2.1. Chemicals

D_2_O (>99%), HEPES powder, calcium chloride pellets, chloroform:phenol:isoamyl alcohol 49.5:49:5:1, chloroform, petroleum ether (boiling point 40–60°C) and rough-type lipopolysaccharide (RaLPS) extracted from *Escherichia coli* strain EH100 were purchased from Sigma–Aldrich and used without further purification. Tail deuterated (D = 62) dipalmitoylphosphatidylcholine (d_62_DPPC) and 1-palmitoyl-2-oleoyl-*sn*-*glycero*-3-phosphocholine (POPC) were purchased from Avanti lipids and used without further purification.

### 2.2. Preparation of asymmetric bilayers via LB/LS depositions

**Figure 2:**
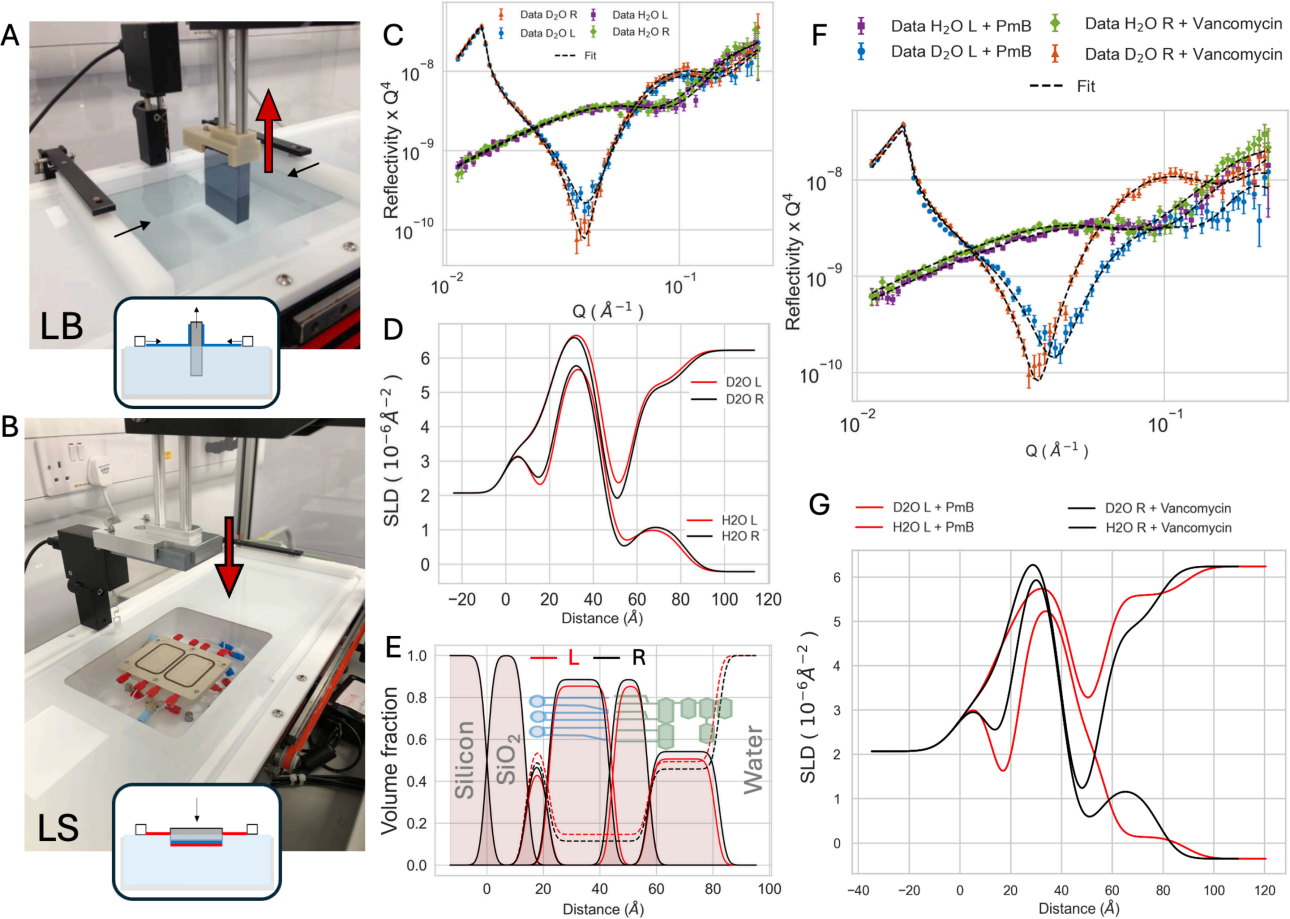
Asymmetric lipid bilayers for antibiotic research using the P1 prototype. Setup for the preparation of asymmetric lipid bilayers via sequential LB (A) and LS (B) depositions; insets show a cartoon of the sequential monolayer transfers. NR data and fits for asymmetric dDPPC/LPS bilayers measured in the two compartments of the cell (labelled L and R) (C), and corresponding SLD profiles (D). Volume fraction distribution of the bilayer components (solid lines) and water (dashed lines) in the two samples with bilayer schematics overlaid (E). Roughness reduced to 2 Å for clarity. Plot with original roughness is shown in Fig. S3. NR data (F) and corresponding SLD profiles (G) after the addition of vancomycin (black) and polymyxin B (red) antibiotics in one of the two compartments.

Asymmetric lipid bilayers containing d_62_DPPC in the inner leaflet and LPS in the outer leaflet were prepared using sequential Langmuir–Blodgett/Langmuir–Schaefer (LB/LS) depositions as described extensively in previous papers using the equipment available at ISIS (Clifton *et al.*, 2019*a* ▸; Clifton *et al.*, 2013 ▸; Paracini *et al.*, 2018 ▸; Paracini *et al.*, 2022 ▸). Briefly, a monolayer of d_62_DPPC was spread on a Milli-Q subphase in a Langmuir trough from a 1 mg ml^−1^ solution in chloroform, compressed to 37 mN m^−1^ and transferred on a submerged clean silicon substrate using LB deposition at 3 mm min^−1^ withdrawal speed. LPS, dissolved in phenol:chloroform:petroleum ether 2:5:8 at 1 mg ml^−1^ , was spread on a Milli-Q subphase cooled to 10°C containing 5 m*M* CaCl_2_, compressed to 37 mN m^−1^ and transferred by LS onto the silicon substrate coated with d_62_DPPC. The substrate was then lowered in the well of the trough onto the P1 solid–liquid cell previously prepared to house the substrate and sealed under water in the well (Figs. 2A and 2B).

### 2.3. Cell assembly

P2_H_ and P2_V_ cell components, except for the silicon crystal, were cleaned by sonication in ethanol followed by sonication in water. The cells were then connected to Teflon tubing using high-performance liquid chromatography (HPLC) fittings, and the flow path was rinsed with ethanol followed by water. The cells were placed flat in a laminar flow hood where Milli-Q water was injected in each tubing connection to remove air bubbles until a pool of water with a ∼2 mm meniscus formed on each compartment, delimited by an O-ring. The freshly cleaned hydrophilic substrate was then carefully aligned and lowered on top of the cell to avoid trapping air bubbles in the assembled cells. The cell was sealed by screwing the backplate in place, ensuring an even distribution of pressure between the screws, and fixed to the sample stage using three-point magnetic kinematic mounts (Thorlabs, USA).

### 2.4. Preparation of gold-coated substrates

Gold-coated silicon substrates were prepared by sputter coating at the ILL (Cozzolino *et al.*, 2024 ▸). A titanium layer of 5 nm was used as a binder between the native silicon oxide and a ∼25 nm layer of gold. Before mounting the substrate in the solid–liquid cell, the surface was cleaned in a ProCleaner Plus UV/ozone cleaner (BioForce Nanosciences). The crystal was left in the ozone cleaner for 30 min and then rinsed under a stream of Milli-Q water. The cleaning was repeated twice before assembling the substrate in the P2_H_ solid–liquid cell.

### 2.5. Preparation of supported lipid bilayers via vesicle fusion

Supported lipid bilayers (SLBs) were prepared via vesicle fusion. Briefly, a chloroform solution of POPC was aliquoted in a glass vial and dried under nitrogen to yield a lipid film of 1 mg. The lipids were dispersed in 1 ml of Milli-Q water, vortexed, sonicated in a water bath for 10 min and tip sonicated with 5 s on/off cycles at 30% amplitude using an FB120 tip sonicator (Fischer Scientific). The vesicle suspension was diluted to 0.1 mg ml^−1^ in a 2 m*M* solution of CaCl_2_ and injected in the pre-assembled *P*2_V_ solid–liquid cell containing a clean silicon substrate.

### 2.6. Reflectivity measurements

All reflectivity measurements were performed by under-illuminating the area of each compartment. The lengths of the compartments reported below are the centre-to-centre distance between the O-ring grooves.

Measurements on the OFFSPEC reflectometer (Dalgliesh *et al.*, 2011 ▸) at ISIS were performed at two incidence angles of 0.7° and 2.0° with neutron wavelengths between 2 and 14 Å. The beam footprint was set to 35 by 30 mm in order to under-illuminate a compartment of size 54 mm (along the beam) by 35 mm of the P1 prototype. Measurements were performed for a total current of 5 and 30 µA for the first and second angle, respectively, corresponding to ∼8 and ∼50 min under normal accelerator operations with a d*Q*/*Q* resolution of 4%.

Measurements on the FIGARO reflectometer (Campbell *et al.*, 2011 ▸) at ILL were performed at two incidence angles of 0.7° and 3.0° with neutron wavelengths between 2 and 20 Å. The beam footprint was set to 26.2 by 26.6 mm in order to under-illuminate the compartment of size 44 mm (along the beam) by 35 mm for the P2_H_ cell. Measurements were performed for 5 and 15 min for the first and second angles, respectively, with a d*Q*/*Q* resolution of 7%.

Measurements on the D17 reflectometer (Saerbeck *et al.*, 2018 ▸) at ILL were performed at two incidence angles of 0.8° and 3.0° with neutron wavelengths between 2 and 30 Å. The beam footprint was set to 35 by 15 mm in order to under-illuminate the compartment of size 44 mm (along the beam) by 20 mm of the P2_V_ cell. For the measurements on D17 a cadmium mask was placed before and after the cells to prevent the divergent beam from over-illuminating the compartments (Fig. S1 in the supporting information). The measurement times for the first and second angle were 5 and 40 min in D_2_O, 30 and 60 min in 38% D_2_O (silicon matched water, SMW), and 10 and 50 min in H_2_O.

For all the NR measurements the samples were characterized under multiple isotopic contrast conditions by connecting the solid–liquid cells to an HPLC pump fed with H_2_O and D_2_O solutions. The asymmetric bilayer was measured in a buffer containing 20 m*M* HEPES pH 7.4 supplemented with 5 m*M* Ca^2+^, whilst the gold-coated substrate and phospholipid SLB were measured in pure H_2_O and D_2_O

### 2.7. Analysis of neutron reflectometry data

NR curves were fitted using the *refnx* software (Nelson & Prescott, 2019 ▸). *Jupyter* notebooks and datasets required to reproduce the analysis are available on Zenodo (Paracini, 2025 ▸). The datasets were analysed using slab models that feature a series of layers representing chemically different regions in the sample.

The asymmetric bilayer was modelled as a series of four slabs representing, in order, moving away from the silicon substrate and its silicon oxide layer, (i) the phospholipid head group, (ii) the phospholipid tails, (iii) the LPS tails and (iv) the LPS head group. A single hydration value was used for the lipid tails assuming that water present in this region is due to defects that span the whole lipid bilayer. Each one of the four slabs modelling the head groups and tail regions is described by layer thickness, roughness and material scattering length density (SLD) values which can be used to reconstruct the volume fraction profile of the components across the interface as well as the volume fraction of water penetrating in the different regions of the sample. The model was set up to minimize the number of free parameters fitted by sharing common parameters for multiple datasets. Parameters related to the silicon substrate such as roughness and SiO_2_ layer thickness were shared between the models for the samples measured on the two sides of the cell. The roughness of the bilayer was assumed to propagate from one leaflet to the other and was therefore constrained to vary uniformly across the four regions of the bilayer and described by a single value. This approach is typical in contrast variation NR, where the sample is measured multiple times against a background solution composed of different D_2_O/H_2_O mixtures; the resulting datasets are constrained to share the same values of SLD, layer thickness and roughness values, but the datasets are allowed individual values for the SLD of the aqueous solution (Clifton *et al.*, 2019*a* ▸). The SLD is given by
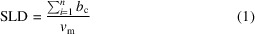
where *b*_c_ is the coherent scattering length of the *i*th atom in a given molecule and *v*_m_ is the molecular volume.

The gold-coated silicon sample was modelled as a series of three slabs, representing SiO_2_, titanium and gold layers. For each layer the SLD was fixed to the calculated value of 3.47, −1.92 and 4.66 × 10^−6^ Å^−2^, respectively, and the thickness and interfacial roughness were left to vary.

The POPC SLB was modelled as two symmetric leaflets where each head group and pair of tails of the phospholipid molecule were modelled using a single area-per-molecule parameter to parametrize the leaflet on the basis of molecular volumes taken from values published by Armen *et al.* (1998 ▸).

Parameter estimation was first performed using the differential evolution algorithm. Markov chain Monte Carlo (MCMC) sampling was then employed to obtain the posterior distribution of the model parameters, providing 68% confidence intervals of the obtained values. The marginalized posterior distributions obtained from MCMC sampling provide a detailed view of the parameter space.

## 3. Results

### 3.1. Cell design rationale

Three different types of cells, based on existing designs, were built to test the concept of a twin-compartment solid–liquid cell: an initial prototype and two versions of a more refined cell based on the modular design proposed by Rennie *et al.* (2015 ▸). The initial prototype (P1) is a basic adaptation of a large solid–liquid cell design that houses a silicon block of surface area 60 × 80 mm^2^. The long side of each compartment is oriented parallel to the short side of the silicon crystal so that the neutron beam illuminates the sample along the short side of the substrate (Fig. 1 ▸B). This cell was tested on the OFFSPEC reflectometer at ISIS to study an asymmetric lipid bilayer prepared by Langmuir–Blodgett and Langmuir–Schaefer depositions. The two other cell prototypes were designed with refined features for two different types of reflectometers: a cell housing a 50 × 80 mm^2^ substrate optimized for horizontal collimated reflectometers (*e.g.* Freia at ESS, FIGARO at ILL, INTER at ISIS), named P2_H_ (Fig. 1 ▸C), and a smaller cell housing a 50 × 50 mm^2^ substrate optimized for vertical reflectometers exploiting divergent neutron beams (Estia at ESS and D17 at ILL), referred to here as P2_V_ (Fig. 1 ▸D). An important aspect to consider when using solid–liquid cells in a vertical setup is the different density of D_2_O and H_2_O. It is necessary to ensure that, when the density of the injected liquid is higher than that of the liquid inside the cell, the denser solution is added from the bottom of the cell, whilst when exchanging a denser liquid with a less dense solution the latter must be injected from the top to avoid inhomogeneous exchange. P2_V_ was designed with these aspects in mind, and the inlets and outlet ports for the solution exchange are placed so that ports on the same side of the cell feed the two compartments from the same side, making it easy to distinguish injections from the top or from the bottom when the cell is vertically oriented. The arrangement of the flow channels of P2_V_ can be seen in the transparent polycarbonate version (Fig. S2). The cells were manufactured in both polyether ether ketone (PEEK) and polycarbonate. The former is the standard material used for solid–liquid cells as it combines chemical resistance with good mechanical properties for manufacturing. The latter displays good chemical resistance and has the advantage of being transparent to visible light, allowing for visual inspection of the sample surface and sample irradiation experiments on non-transparent substrates.

### 3.2. P1 – application to a sample prepared by LB/LS depositions

The P1 prototype was used to compare the effect of antibiotics on asymmetric lipid bilayers that mimic the surface of Gram-negative bacteria and are used in antimicrobial research (Clifton *et al.*, 2016 ▸; Paracini *et al.*, 2018 ▸; Paracini *et al.*, 2025 ▸; Gong *et al.*, 2021 ▸). The model membranes contain deuterated phospholipids (d_62_DPPC) in the inner leaflet and hydrogenous LPS in the outer leaflet and are assembled using Langmuir–Blodgett and Langmuir–Schaefer depositions (see Section 2.2 and Figs. 2 ▸A and 2 ▸B). When the procedure is carried out using a two-compartment cell, this approach allows preparation of two samples in the same amount of time as it takes to make a single larger sample with a standard single-compartment cell, which typically takes several hours to perform for a skilled user, therefore significantly speeding up the sample preparation process. NR measurements were performed on the OFFSPEC reflectometer (horizontal sample geometry) to characterize the samples on both sides of the compartment. The resulting reflectivity curves, measured in both H_2_O and D_2_O solution contrasts on both compartments, show comparable reflectivity profiles with small variations, mainly visible in the minima of the D_2_O profile (Fig. 2 ▸C). To quantitatively compare the data obtained from the two compartments, the reflectivity was fitted using *refnx* by constraining the substrate parameters related to silicon and the native silicon oxide layers to be common parameters between the fits of the samples on the two sides of the cell, and leaving the parameters related to the lipid bilayer free to vary independently for the two samples within reasonable bounds. The SLD and volume fraction profiles confirm the samples’ structural similarity with only minor variations (Figs. 2 ▸D, 2 ▸E and S3). An in-depth comparison of the posterior distribution obtained from MCMC sampling of the fits can be found in the supplementary material (Fig. S4). The results of the fits show the expected asymmetric structure measured on both compartments, which can be further appreciated by comparing the volume fraction distribution of the bilayer components at the interface (Fig. 2 ▸E). After measuring the two compartments in two contrasts, the cell was heated to 37°C and two different antibiotic molecules were injected on either compartment. The right compartment was treated with vancomycin whilst the left compartment was treated with polymyxin B (PmB) (Fig. 2 ▸F). PmB is active against Gram-negative bacteria and is known to penetrate and disrupt the asymmetric bilayer structure at 37°C (Paracini *et al.*, 2018 ▸; Paracini *et al.*, 2025 ▸). Van­comycin is active against Gram-positive bacteria and tagets cell wall synthesis and was therefore used here as a con­trol. As expected, PmB caused large-scale effects on the corresponding lipid bilayer, altering the lipid distribution, whilst vancomycin did not produce any major effect on the outer membrane model structure (Fig. 2 ▸G). Overall, this proof-of-concept experiment showed that the twin-compartment solid–liquid cell provides significant advantages when used to study LB/LS samples, cutting sample preparation time in half and providing a reliable platform for comparative membrane interaction studies.

### 3.3. P2_H_ – gold-coated 80 × 50 mm^2^ substrate

**Figure 3:**
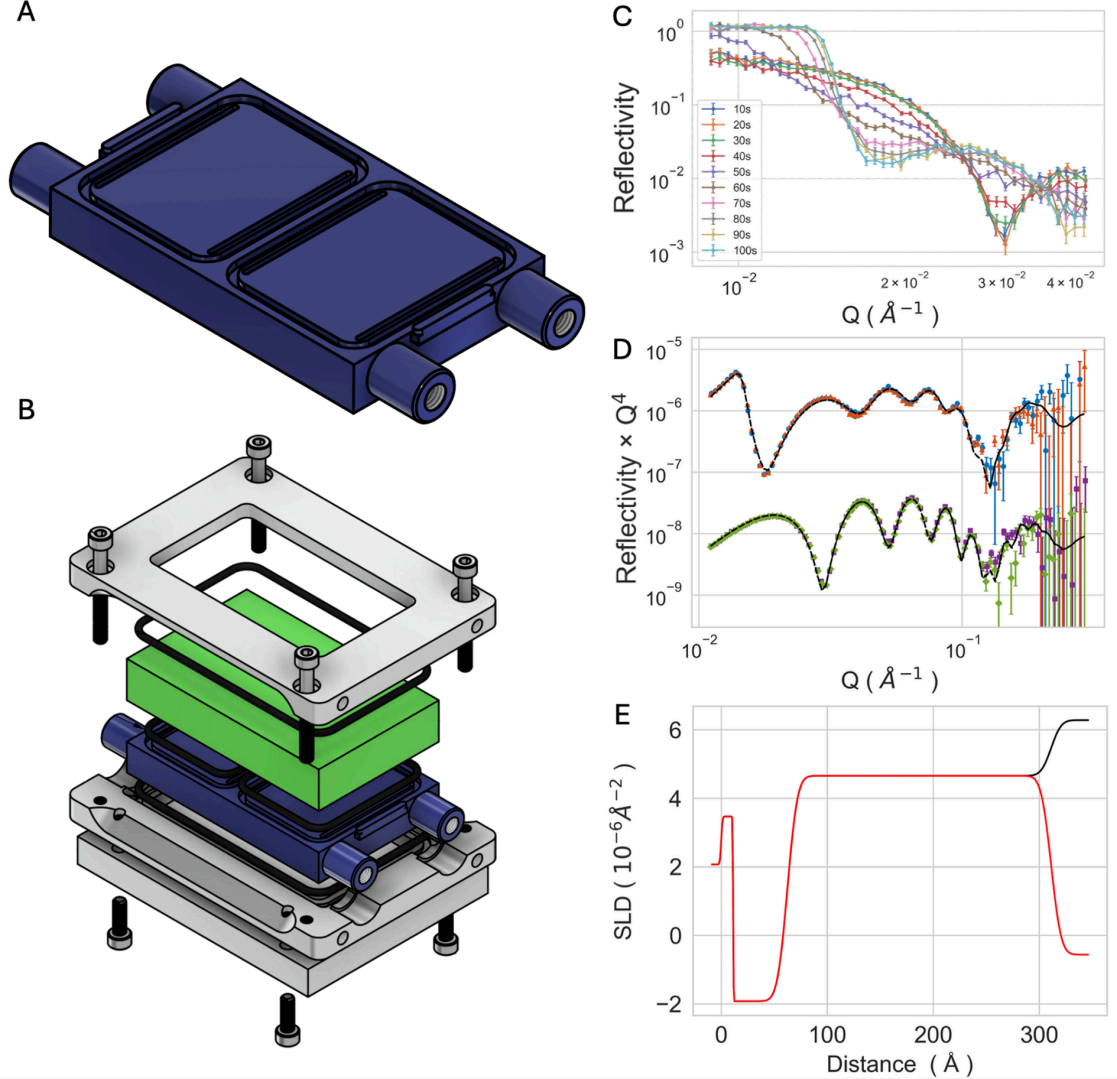
Measurements of a gold-coated silicon crystal using the P2_H_ cell. Design of the cell front piece (A) and exploded assembly (B). Reflectivity measurement during exchange from D_2_O to H_2_O at a flow rate of 1 ml min^−1^ (C). Neutron reflectivity from the gold-sputtered substrate measured on the two compartments in D_2_O (orange and blue) and in H_2_O (green and purple, data offset vertically for clarity) (D), and corresponding SLD profiles (E).

The P2_H_ cell (Figs. 3 ▸A and 3 ▸B) is the twin-compartment cell that houses the standard 50 × 80 mm^2^ sized silicon blocks in use at ISIS and ILL. It was tested on the FIGARO reflectometer (horizontal sample geometry) to characterize a silicon substrate sputter-coated with a ∼25 nm layer of gold. In addition to the gold coating, a thin ∼5 nm titanium layer acts as binder between the silicon oxide and the gold layer to increase the stability towards lamination. Gold-coated substrates can be functionalized with thiol-terminated mol­ecules to produce a range of interfaces. They are, therefore, a popular substrate in surface science and are often used in NR studies (Hughes *et al.*, 2014 ▸; Lu *et al.*, 2007 ▸; Clifton *et al.*, 2019*b* ▸; Skoda *et al.*, 2009 ▸). Whilst P1 delivers solution to the compartment through a series of holes distributed along the long side of the compartment, similarly to current solid–liquid cells in use at ILL and ISIS (Welbourn & Clarke, 2019 ▸), P2_V_ and P2_H_ have a different design of the channels with a single hole positioned at one end of a groove that runs along the compartment. We tested the volume of solution required to completely exchange the content of the cells from H_2_O to D_2_O with this flow path design by looking at the evolution of the reflectivity curve in the region of total reflection, measuring the first angle during the solvent exchange. The reflectivity was measured in 10 s acquisitions whilst pumping solution using an HPLC pump with a flow rate of 1 ml min^−1^ (Fig. 3 ▸C). The solution exchange was complete within 100 s from when the solution reached the compartment (thus excluding the dead volume of tubing in the pump system), requiring ∼1.7 ml to fully exchange the content of the cell from one solution to the other. The reflectivity of the gold layer was then measured over the full *Q* range to characterize the structure of the interface in H_2_O and D_2_O in both compartments. The curves overlap over the measured *Q* range, confirming that the substrates in the two compartments are identical, and were therefore fitted using the same parameters (Fig. 3 ▸D). The data were analysed using *refnx* to obtain the parameters of the interfacial layers, which yielded layer thickness and roughness values for the silicon oxide, titanium and gold layers represented in the SLD profile of Fig. 3 ▸E. The fits yielded thickness values of 11.2 ± 0.2, 51.2 ± 0.1 and 248.6 ± 0.1 Å for silicon oxide, titanium and gold layers, respectively, in excellent agreement with the nominal 50 Å Ti and 250 Å Au requested from the manufacturer. The preparation of sputter-coated samples is not only time consuming but also an expensive process and requires high skill and specialist equipment to obtain homogeneous layers over large surfaces. The twin-compartment solid–liquid cell provides an effective way to optimize the use of these types of substrates in NR experiments.

### 3.4. P2_V_ – vesicle deposition on 50 × 50 mm^2^ silicon substrate

**Figure 4:**
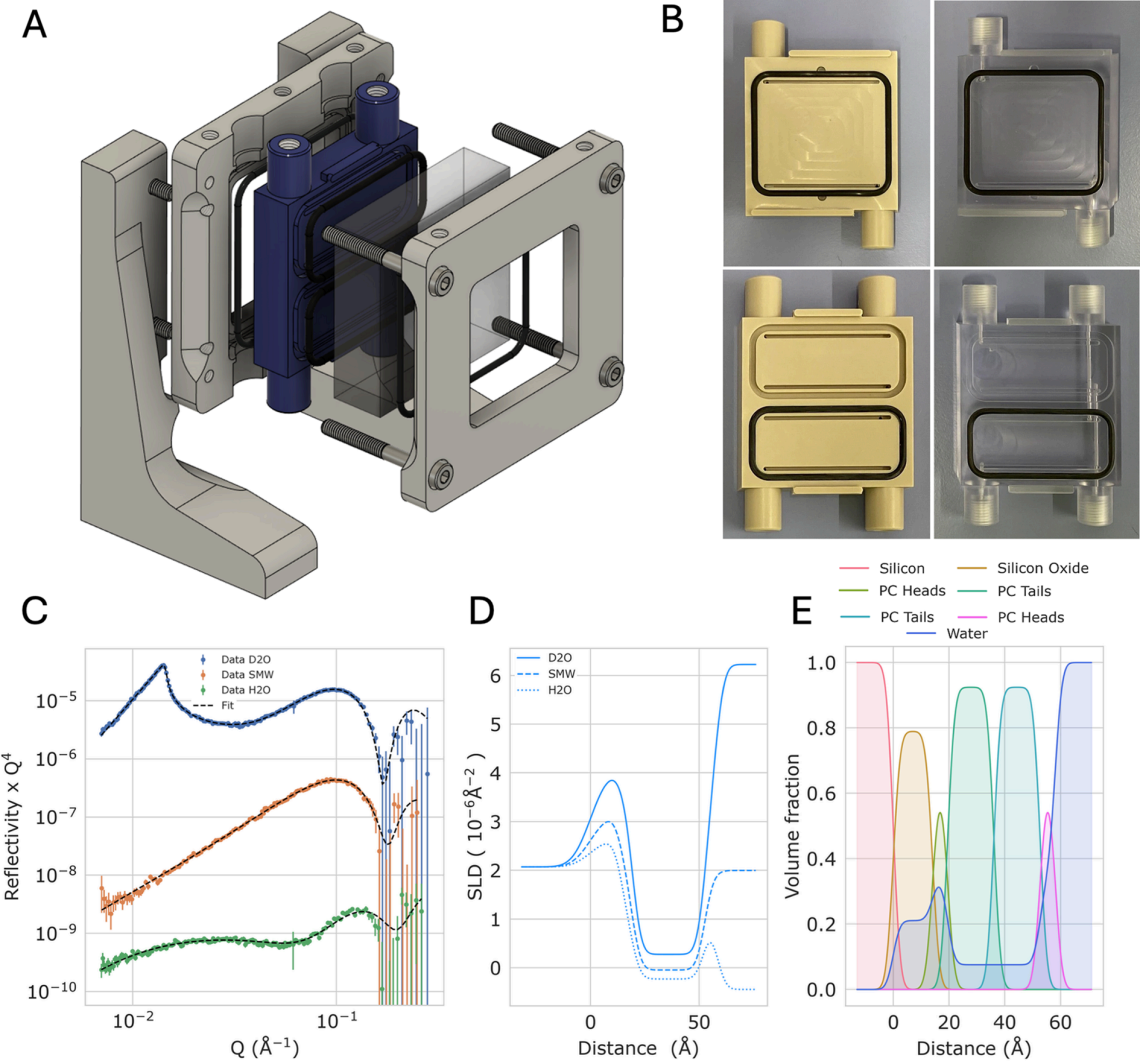
Measurements of a lipid bilayer using the P2_V_ cell. Exploded assembly of the P2_V_ cell (A); compatible front pieces with the cell frame including single- and two-compartment models in the PEEK and transparent polycarbonate versions (B). Neutron reflectivity measurement of a POPC lipid bilayer in one of the two compartments of P2_V_ (PEEK version) in D_2_O, SMW and H_2_O contrasts (C), corresponding SLD profiles (D), and volume fractions of the components across the interface (E). Reflectivity data are offset vertically for clarity; non-scaled plots, together with in-depth error analysis and a table of values, are shown in Fig. S7.

The P2_V_ cell (Figs. 4 ▸A and 4 ▸B) is the cell optimized for reflectometers that can exploit a divergent or focused neutron beam and are therefore capable of measuring smaller surface areas. This is typically required for hard-matter samples, which can often be smaller than 1 cm^2^. The cell was designed to fit the beam characteristics of the Estia reflectometer at ESS (vertical sample geometry), which exploits the Selene guide concept to focus the neutron beam on the sample (Stahn & Glavic, 2016 ▸). The focused beam produced by Estia should reach a maximum width of 1 cm in the vertical direction, which makes it possible to build a twin-compartment cell that houses a 50 × 50 mm^2^ substrate, yielding two compartments of 20 × 44 mm^2^. The cell was produced in two versions, one in PEEK and one in polycarbonate. Polycarbonate has the advantage of being transparent whilst maintaining good chemical stability, which makes it easy to spot formation of bubbles on the substrate surface. This is a common problem during solid–liquid NR measurements, as dissolved oxygen in buffers or air introduced during liquid injections can generate air pockets on the substrate surface. These alter the reflectivity of the sample, replacing part of the solid–liquid interface with an air–solid interface. This in turn has a negative impact on data analysis, which generally assumes a homogeneous sample across the illuminated area. Unlike Estia, D17 does not have focusing optics like the Selene guides but rather exploits a large guide and two slits to create a divergent beam (Saerbeck *et al.*, 2018 ▸). Therefore, to reduce background and prevent the divergent beam from over-illuminating the sample, a cadmium mask with a height of 15 mm was placed before and after the cell (Fig. S1), effectively acting as an additional slit defining the beam height. With the cadmium mask in place the sample was aligned with the centre of the top compartment in the beam, and different apertures of the vertical gap of slit 3 (the slit placed just before the sample on D17) were tested with the goal of understanding when the beam would start to go through the second nearby aperture of the cadmium mask. This was performed to determine the size of slit 3 to use during measurements. The compartment being measured was filled with H_2_O whilst the nearby compartment contained D_2_O. The reflectivity looked as expected for a silicon/H_2_O interface up until an opening of 25 mm. Opening the slit to 30 mm showed the appearance of the critical edge coming from the beam illuminating the nearby compartment filled with D_2_O (Fig. S5). For the measurements we selected a conservative aperture of 16 mm, significantly below 25 mm in order to be safely below the limit. We note that the slit can be opened to gain significantly more intensity. As done for P2_H_, the volume of solution required to exchange contrast in the cell was tested by monitoring the reflectivity during solvent exchange carried out at 1 ml min^−1^, which showed complete exchange in under 100 s and 1.7 ml of solution (Fig. S6). We used the cell to prepare an SLB via vesicle fusion in one of the two compartments and measured the sample in three solution contrasts, H_2_O, D_2_O and 38% D_2_O (SMW) (Fig. 4 ▸C), whilst the second compartment was left filled with D_2_O during the measurements. Data were fitted using a symmetric leaflet model where each head group and pair of tails in the phospholipid molecules were constrained to fit a single area-per-molecule value. The resulting SLD and volume fraction profiles show the expected features of a high-coverage POPC lipid bilayer with an area per molecule of ∼59 Å^2^ and a coverage of >90% (Figs. 4 ▸D, 4 ▸E and S7). The same lipid bilayer was prepared using the transparent polycarbonate front piece and measured in D_2_O to compare the level of background noise obtained. Incoherent scattering from hydrogen is one of the main contributions to background noise in NR measurements. The hydrogen content of polycarbonate is higher than that of PEEK, with 14 hydrogen atoms per unit monomer versus 8, but with a marginally lower density of ∼1.2 versus ∼1.3 g cm^−3^. Despite the differences in hydrogen content we observed no significant difference in the background levels in the measurements performed with the two cells (Fig. S8), which is probably because for macroscopic liquid trough thicknesses most of the background comes from the bulk liquid itself (Hoogerheide *et al.*, 2022 ▸).

## 4. Discussion

NR has emerged as a powerful structural technique to study the architecture of complex soft-matter and biological interfaces, due to a combination of fundamental properties that set neutrons apart from X-rays: (i) lack of radiation damage; (ii) high penetration to access buried interfaces through complex sample environments; and (iii) hydrogen sensitivity and protium/deuterium contrast variation. This makes neutrons an ideal probe to study phenomena in soft matter and biology at solid–liquid interfaces. Solid–liquid cells are therefore an essential part of the sample environment of a neutron reflectometer and have not conceptually changed since the first experiments carried out on this type of interface (Lee *et al.*, 1989 ▸). In the meantime, the performance of neutron reflectometers has significantly improved thanks to instrument upgrades and developments in neutron transport technology, increasing the number of measurements that can be carried out within one allocated beam time. What has not changed, however, is the number of samples that can be mounted on the sample stage of a reflectometer, which is constrained by the size of the stage and the size of the solid–liquid cells. An additional current-limiting factor is sample preparation time for short-lived samples that require *ex situ* preparation immediately before the beam time begins. These include lipid bilayers prepared by LB/LS, which require several hours of careful supervised work for each sample (Hughes *et al.*, 2002 ▸; Hughes *et al.*, 2014 ▸; Clifton *et al.*, 2013 ▸; Fragneto *et al.*, 2012 ▸; Fragneto *et al.*, 2001 ▸; Paracini *et al.*, 2018 ▸; Paracini *et al.*, 2025 ▸; Paracini *et al.*, 2020 ▸; Lakey *et al.*, 2022 ▸), spin-coated samples, including oil films for measuring the solid–oil–water interface (Campana *et al.*, 2012 ▸; Campana *et al.*, 2015 ▸), chemically grafted polymer layers (Gresham *et al.*, 2021 ▸; Johnson *et al.*, 2025 ▸; Robertson *et al.*, 2024 ▸) and self-assembled organic monolayers (Hughes *et al.*, 2002 ▸; Gavutis *et al.*, 2025 ▸; Clifton *et al.*, 2019*b* ▸). As neutron flux increases, users at neutron facilities need to be able to prepare enough samples, within a reasonable amount of time, to fully exploit allocated beam time and optimize the arrangement of samples on the sample stage to take advantage of automation and reduce dead sample change and alignment times. The twin-compartment solid–liquid cells described here build on the large-surface legacy of NR samples and take advantage of current large substrates to optimize several aspects of solid–liquid measurements by splitting the substrate into two independent samples. In addition, the modular design of the cells, based on the concept by Rennie *et al.* (2015 ▸), allows for easy conversion of the sample environment from the single compartment to twin-compartment setup by simply swapping one component of the assembly whilst maintaining the same substrate size (Fig. 4 ▸B). This has the practical advantage that, at ESS, with a 50 × 50 mm^2^ substrate, a standard single-compartment cell can exploit the full beam footprint on Freia (maximum width ∼35 mm), while the twin-compartment cell allows the same substrate to be optimized for Estia’s focused beam (maximum width ∼10 mm) by splitting the surface into two independent areas.

We tested three prototypes on three different neutron reflectometers – OFFSPEC at ISIS, and FIGARO and D17 at ILL – and showed that cells based on this concept enable experiments to be carried out on two samples prepared on a single substrate under standard measurement conditions and on current instrumentation. On OFFSPEC we ran a typical NR interaction study, where two compounds, in this case two antibiotics, are tested for their ability to interact with a model of the bacterial membrane, a very active and growing field of research in NR (Caselli *et al.*, 2024 ▸; Paracini *et al.*, 2022 ▸; Lakey *et al.*, 2022 ▸). The twin-compartment cell was shown to provide significant advantages for these applications. Sample pre­pa­ra­tion, which in this case involves LB/LS depositions to form asymmetric lipid bilayers, was sped up by a factor of 2 compared with the standard approach, and the characterization showed comparable samples in the two compartments of the cell (Figs. 2 ▸C, 2 ▸D, 2 ▸E, S3 and S4). This allowed for side-by-side comparison of the effects of the antimicrobial compounds (Figs. 2 ▸F and 2 ▸G). Given the temperature dependence of polymyxin’s antibiotic effects, an additional advantage in this specific case is the homogeneous temperature across the two compartments. As they share a common heating circuit, the variability in temperature fluctuations that can occur between separate samples is reduced. While LB/LS depositions require specialized equipment and involve more elaborate workflows compared with self-assembly, they remain a unique tool for achieving precise control over the composition and molecular packing of multilayered samples. Specifically, they are cur­rently the only approach capable of yielding highly asymmetric lipid bilayers, such as those investigated here, which are essential for mimicking key biological barriers like the Gram-negative bacterial outer membrane (Nikaido, 2003 ▸; Paracini *et al.*, 2022 ▸). Furthermore, for sophisticated lipid mixtures such as purified natural extracts, LB/LS depositions provide a reliable alternative when self-assembly protocols fail to produce stable bilayers for NR experiments (Corucci *et al.*, 2025 ▸). Solid–liquid cells that significantly reduce the time required for LB/LS sample preparation therefore expand the practical toolkit of sample environments available to the NR community. More broadly, the twin-compartment architecture increases experimental throughput on existing reflectometers by enabling two independent measurements on a standard substrate, reducing preparation and alignment times while remaining compatible with current sample changers. In addition, it facilitates side-by-side control or repeat measurements on the same substrate, which makes comparative studies and reproducibility checks easier. While control experiments can also be performed on separate substrates using standard solid–liquid cells, different substrates can differ in roughness or other interfacial properties that influence the measured reflectivity. Using a single substrate for both the experiment and its control reduces this source of variability and increases the reliability of the comparison.

As the P1 cell is a very basic adaptation from a single compartment to a two-compartment system, the design of the liquid exchange was not optimized, and the flow path lies along the beam path because the cell has to be turned 90° from its original orientation. The flow path design and performance were optimized and tested in the next versions of the prototype, P2_V_ and P2_H_. This allowed for fast and efficient exchange of the subphase in both models, with full compartment exchange complete within 100 s at 1 ml min^−1^ with <1.7 ml of solution needed (Figs. 3 ▸C and S6). P2_H_ was used to measure a gold-coated silicon block (a type of sample that is often used for experiments on functionalized surfaces) showing identical surfaces on both sides of the cell (Figs. 3 ▸D and 3 ▸E). This optimizes the use of sputter-coated samples which are a versatile substrate of limited availability, often used in NR experiments. P2_V_ was tested in a PEEK and a polycarbonate version for the study of self-assembled supported lipid bilayers formed by vesicle fusion. The samples were measured with a divergent beam on D17, which grants access to smaller samples compared with traditionally collimated beams. A set of three contrasts was collectively measured in 3 h and 15 min, which is a typical measurement time on high-flux reflectometers for lipid bilayers. The analysis of the data shows well-defined values for the structure of the lipid bilayer, with tight error distributions within large bounds and a reduced χ^2^ value of 1.1 (Fig. S7). The polycarbonate cell showed background levels identical to those of the PEEK version (Fig. S8), with the additional advantage of being transparent. This enables visual inspection of the polished silicon face in the assembled cell, which is highly desirable to ensure the absence of trapped air bubbles on the sample surface.

The P2_H_ and P2_V_ cell designs can be further improved in some important aspects. In the current design, the heat conductivity from the water bath channels is not ideal as the contact area between the heating elements and the substrate is reduced by the presence of an additional o-ring and a central aperture designed for SANS measurements (Rennie *et al.*, 2015 ▸). Exchanging the open metal back piece with a solid version and eliminating the back o-ring increases the contact area and the heat exchange efficiency. Additionally, the thickness of the water gap can be further reduced to minimize background noise and cell volume, down to a reservoir thickness of 50 µm (Hoogerheide *et al.*, 2022 ▸). Some considerations must be made when going to very thin water reservoirs with regard to wetting issues with hydrophobic surfaces and higher shear forces that can be generated near the injection port during fast solvent exchange, which can locally affect delicate samples such as free-floating lipid bilayers. Decreasing the thickness of the liquid reservoir reduces background scattering from the solution, while it increases how far the transmitted beam penetrates into the cell body, thereby increasing the relative contribution to the background from the materials used. Cells based on silicon–silicon wafer assemblies with a thin (50–100 µm) liquid gap have been shown to achieve some of the lowest background levels reported for solid–liquid NR, reaching below 10^−8^ (Hoogerheide *et al.*, 2022 ▸). This is at least two orders of magnitude lower than the background levels obtained here (Fig. S9), which are comparable to those for standard PEEK solid–liquid cells used with rectangular substrates. Such low background levels significantly extend the usable *Q* range and therefore improve spatial resolution. They are enabled by a combination of factors, including the use of low-background materials, energy-analysed detection of a polychromatic beam (as implemented, for example, on CANDOR at NCNR) (Maliszewskyj *et al.*, 2018 ▸) and long counting times (17.4 h) in a single contrast (Hoogerheide *et al.*, 2022 ▸). For an exhaustive discussion of background signals and correction strategies in solid–liquid NR, the reader is referred to the articles by Hoogerheide *et al.* (2020 ▸, 2022 ▸). Maximizing the signal-to-noise ratio is key to extending the useful *Q* range; however, the limited nature of beam-time allocations and the need for robust, user-friendly sample environments impose additional constraints on cell design. Silicon remains more brittle and delicate than inherently higher-background but durable alternatives such as PEEK and PC, and the practical gain in signal-to-noise ratio under typical measurement conditions (here ∼1 h per contrast) remains to be evaluated. Although a direct comparison of these approaches under identical instrument conditions is beyond the scope of this work, it would nonetheless be a highly informative exercise. Overall, here we have shown that the new type of sample environment presented opens up new efficient ways of performing solid–liquid NR experiments with higher throughput on current and future neutron reflectometers.

## 5. Summary

We introduce and validate a twin-compartment solid–liquid cell design for neutron reflectometry, which splits a single substrate into two independent measurement areas. Cell prototypes were manufactured and tested, demonstrating that this approach effectively increases sample capacity on the reflectometer stage, reduces alignment and substrate characterization time, and enables true side-by-side comparison experiments on the same substrate.

The flow paths enabled rapid and efficient contrast exchange, achieving complete solvent replacement in under 100 s at 1 ml min^−1^ using ∼1.7 ml. In proof-of-concept measurements, the novel design enabled comparative antibiotic studies on asymmetric bilayers mimicking the Gram-negative outer membrane, reproducing the expected polymyxin B-induced disruption while vancomycin served as a control, and halving LB/LS sample preparation time.

Measurements on sputter-coated Au/Ti substrates confirmed the equivalence between compartments and yielded layer thicknesses consistent with nominal values. High-coverage POPC bilayers formed by vesicle fusion (area per molecule ∼59 Å; >90% coverage) were measured in a single small compartment across three contrasts in ∼3 h 15 min, and a transparent polycarbonate version allowed visual inspection of the substrate surface in the assembled cell without increasing background relative to PEEK.

## Supplementary Material

Supporting information file. DOI: 10.1107/S1600576726000919/roo5007sup1.pdf
