# Supplementary material for: Twin-compartment solid–liquid cells for neutron reflectometry
Source: J Appl Crystallogr. 2026 Mar 8;59(Pt 2):369–80. doi: 10.1107/S1600576726000919 (PMC13060453; doi:10.1107/S1600576726000919)
Supplement: Supplementary file 1 [file j-59-00369-sup1.pdf]

JOURNAL OF APPLIED CRYSTALLOGRAPHY

Volume 59 (2026)

Supporting information for article:

Twin-compartment solid–liquid cells for neutron reflectometry

Nicoló Paracini, Hannah Burrall, Thomas Saerbeck, Philipp Gutfreund, Giovanna Fragneto, Luke A. Clifton, Thomas Arnold and Maritée Cárdenas

1 Supplementary Figures

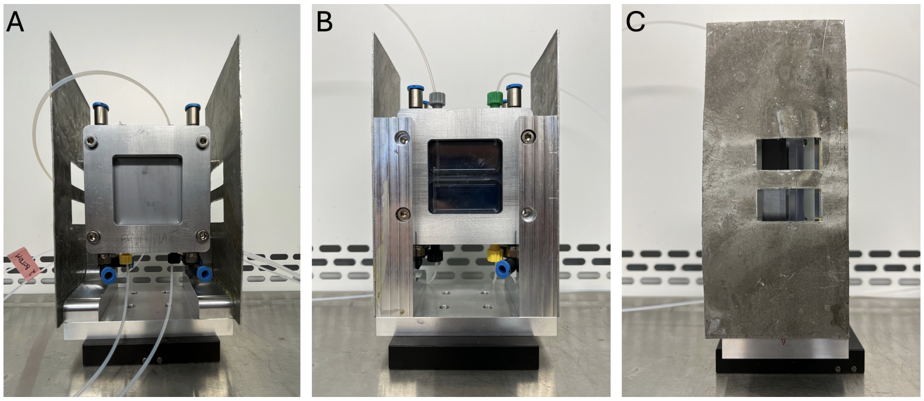

Figure S1: **Photos of the assembled P2$_V$ including the cadmium mask.** The height of the aperture is 15 mm

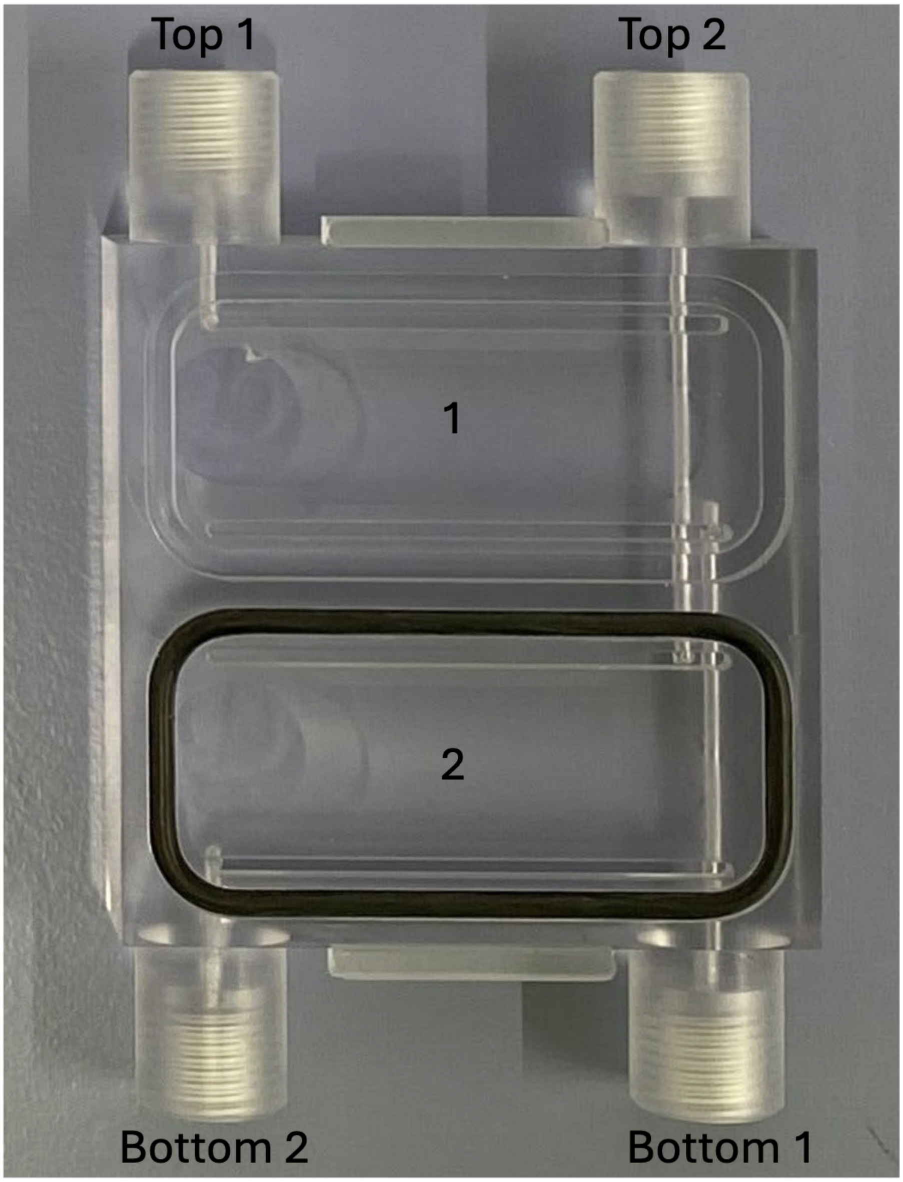

Figure S2: **Flow channels layout in the P2$_V$ prototype.** Inlets placed on the top feed both compartments from the top, and the same for the bottom inlets.

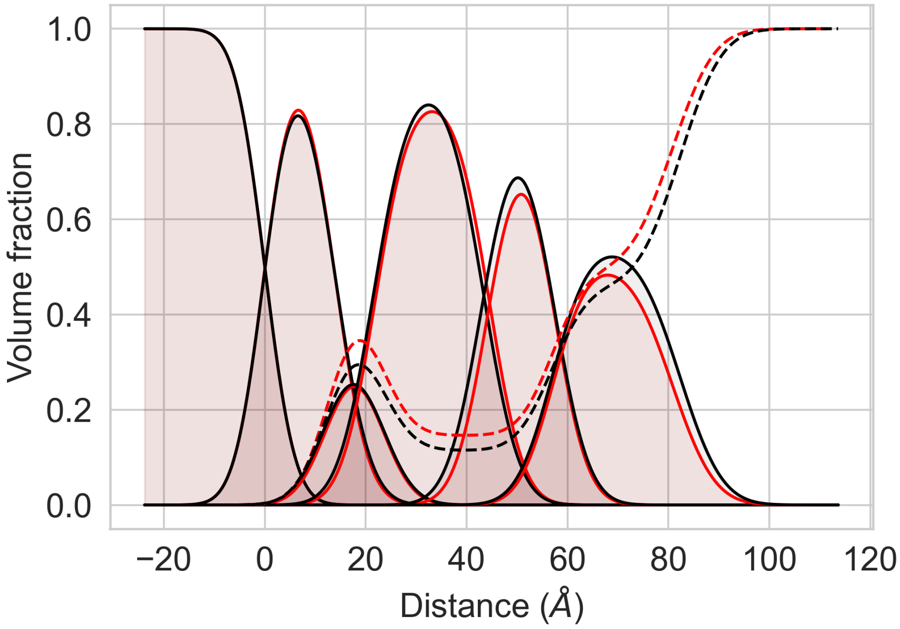

Figure S3: **Comparison of volume fraction profiles plotted with the original roughness.**

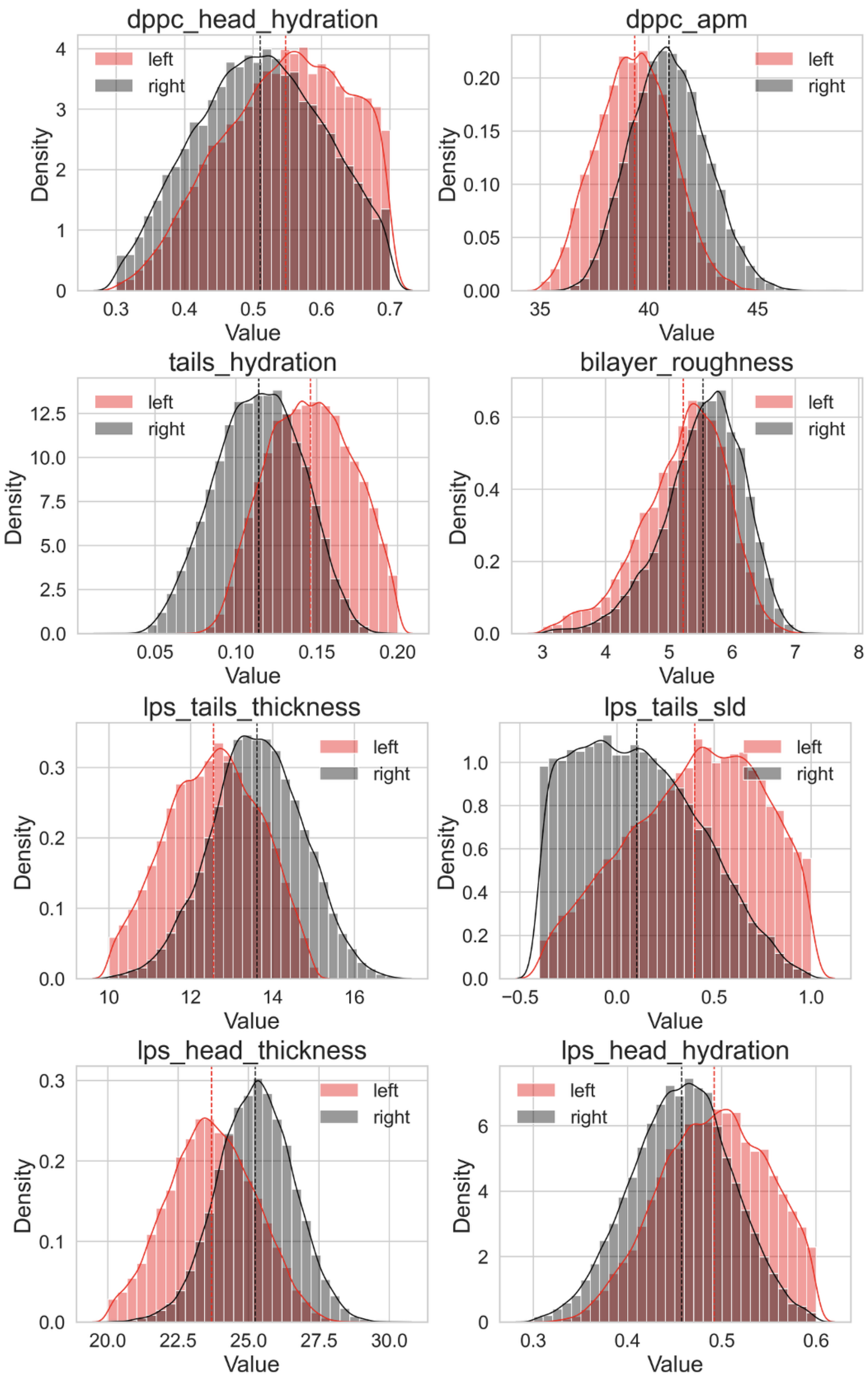

Figure S4: **Marginalised posterior distribution of the parameters obtained from the fits of the asymmetric bilayer measured on the left and right side of P1.**

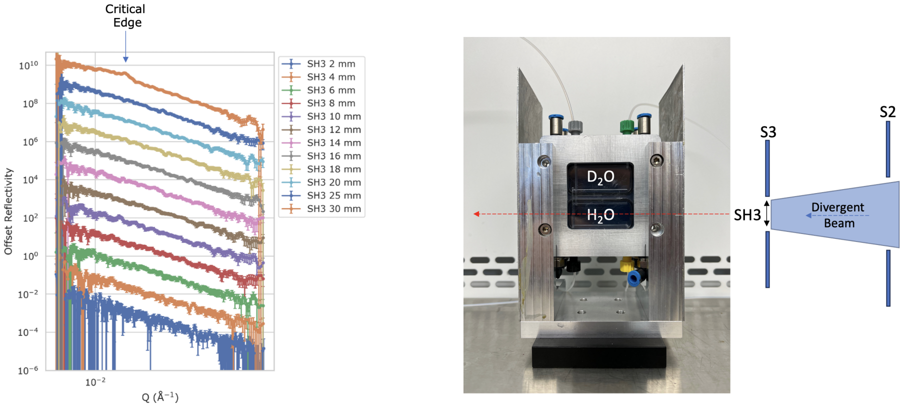

Figure S5: **Testing SH3 aperture on D17.** First angle measurements of the H_2O compartment

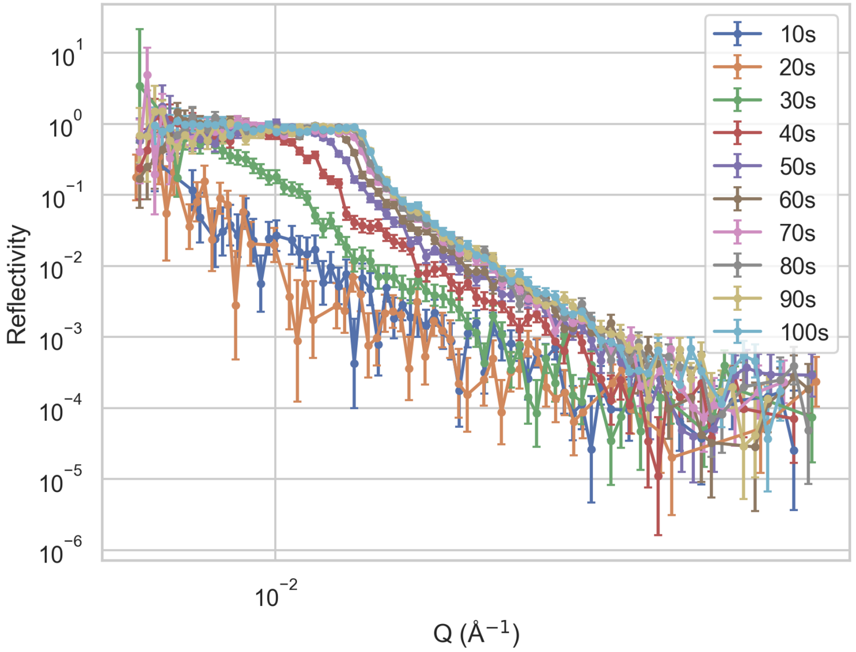

Figure S6: **H_2O to D_2O solvent exchange in $P2_v$** Measurement of a silicon substrate during the exchange of the subphase from H_2O to D_2O

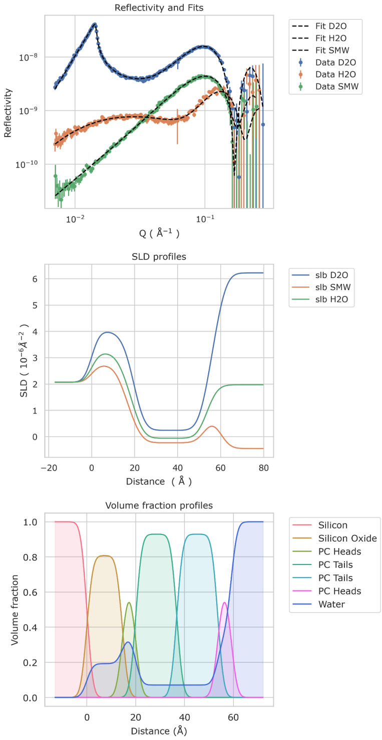

Parameter	Value	StdErr	Bounds
Scale	1.055	0.004	[0.8, 1.2]
SiO2 Thickness	14.497	0.175	[2.0, 30.0]
SiO2 Solvation	0.192	0.005	[0.0, 0.5]
POPC APM	58.180	0.240	[0.0, 100.0]
Heads vol frac	0.650	0.013	[0.0, 1.0]
Tails vol frac	0.929	0.004	[0.0, 1.0]
Roughness	3.895	0.186	[2.0, 7.0]
D2O SLD	6.223	0.004	[6.0, 6.36]
SMW SLD	1.973	0.015	[1.5, 3.0]
H2O SLD	-0.450	0.015	[-0.56, 1.0]
Nr of points	506		
Nr of parameters	10		
Chi²	546.124		
Reduced Chi²	1.101		

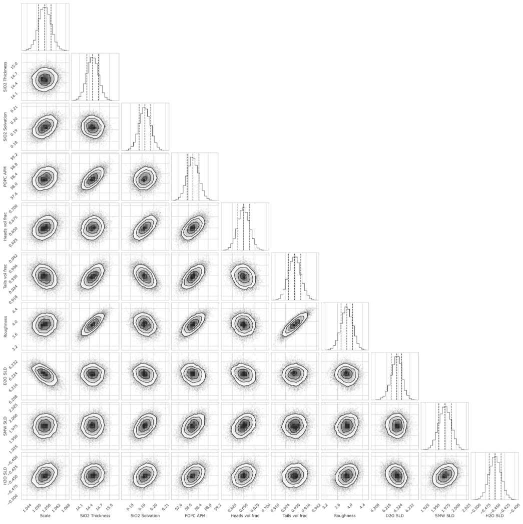

Figure S7: **Summary of the reflectivity analysis for the POPC bilayer measured in the P2$_V$ cell** In addition to the data displayed in the main text the figure shows the parameters values and the corner plot and marginalised posterior distributions from the error analysis on the POPC lipid bilayer.

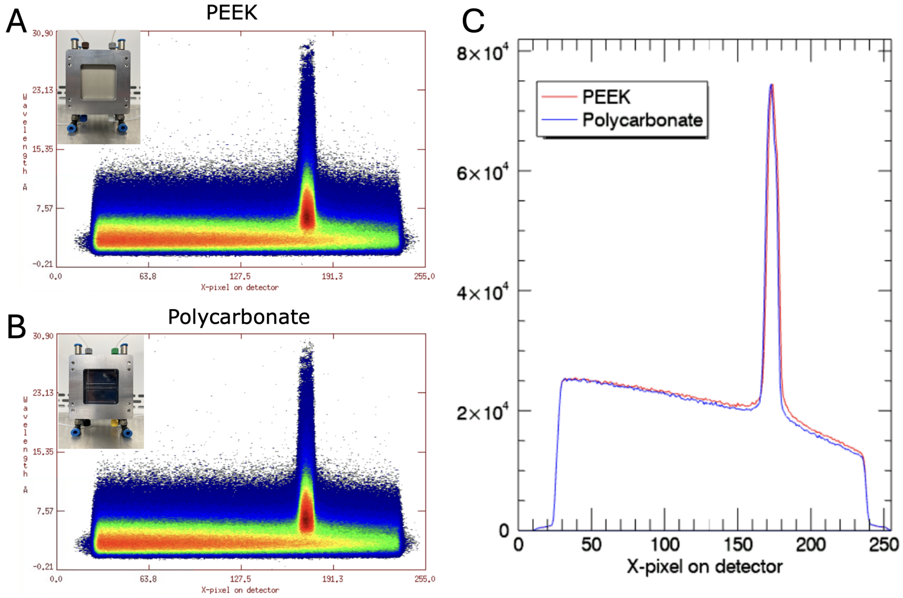

Figure S8: **Background levels of PEEK and polycarbonate versions of P2$_H$** Measurements of the second angle (3.0 degrees) performed on a lipid bilayer of POPC prepared using the PEEK (A) and the polycarbonate (B) front piece of the cell. Panel C shows a comparison of the integrated counts on the detector shown in A and B.

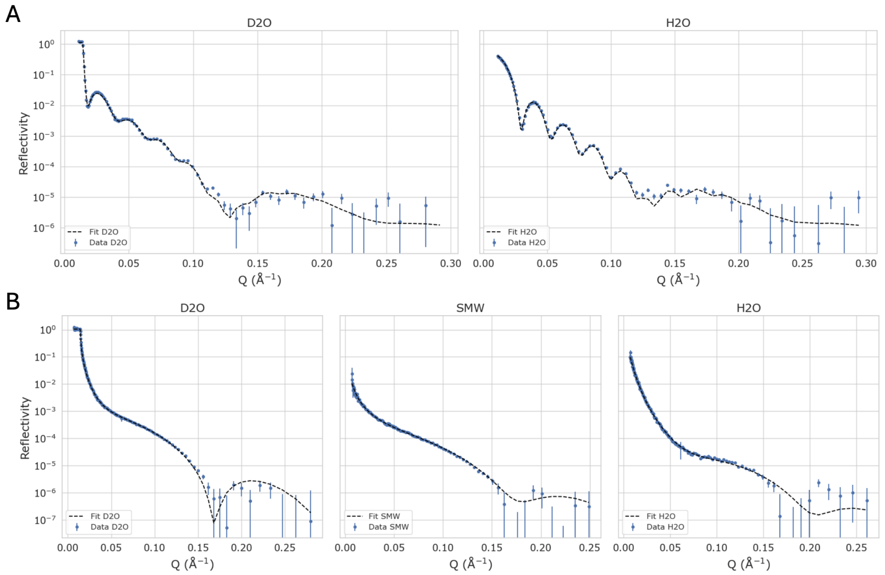

Figure S9: **Reflectivity curves plotted in log-linear scale** Reflectivity curves measured on the gold-titanium substrate in P2$_H$ (**A**) and on the POPC bilayer in P2$_V$ (**B**). Measurement times were 5 and 15 min for the first and second angle respectively in both contrasts shown in **A**, measured on FIGARO and 5 and 40 minutes in D_2O, 30 and 60 minutes in SMW and 10 and 50 minutes in H_2O for the contrasts shown in **B**, measured on D17, as detailed in the methods section.
